# Data in support of association study of the brain-derived neurotrophic factor gene SNPs and completed suicide in the Slovenian sample

**DOI:** 10.1016/j.dib.2015.07.015

**Published:** 2015-07-22

**Authors:** Sandra Ropret, Tomaž Zupanc, Radovan Komel, Alja Videtič Paska

**Affiliations:** a Institute of Biochemistry, Faculty of Medicine, University of Ljubljana, Vrazov trg 2, SI-1000 Ljubljana, Slovenia; b Institute of Forensic Medicine, Faculty of Medicine, University of Ljubljana, Korytkova ulica 2, SI-1000 Ljubljana, Slovenia

**Keywords:** Genotypes, Logistic regression, Inheritance models, Haplotypes, Sliding window procedure

## Abstract

This data article provides the data generated from additional analyses of a genetic association study, where 7 single nucleotide polymorphisms (SNPs) near/within the brain-derived neurotrophic factor (*BDNF*) gene were investigated for an association with completed suicide in Slavic population (Ropret et al., 2015) [1]. One SNP was excluded from the present analyses due to insufficient genotyping rate (rs1491850) and the remaining 6 SNPs (rs7124442, rs10767664, rs962369, rs12273363, rs908867, rs1491851) were analyzed to gain deeper insight into the possible role of these SNPs in the studied phenotype. We present data on logistic regression analyses of: (a) genotypes under four inheritance models, and (b) haplotypes using 2-, 3- and 4-adjacent SNPs sliding window procedure. In both analyses adjustments for potential confounders (age, gender and alcohol dependence syndrome status) were executed. Data may serve as a reference for comparison of the populations with either low or very high suicide rates. The raw genotyping data that could be used in case meta-analyses should be performed may be provided upon request.^1^

**Specifications Table**

**Table:** 

Subject area	*Biology*
More specific subject area	*Neurobiology of psychiatric disorders, particularly completed suicide*
Type of data	*Tables*
How data was acquired	*Software for genetic analysis PLINK v1.07*[2]
Data format	*Analyzed*
Experimental factors	*Genotyping of controls and suicide completers*[1]
Experimental features	*In-depth analysis of genotype and haplotype data, using PLINK v1.07*
Data source location	*Ljubljana, Slovenia, EU*
Data accessibility	*All the data are supplied within this article* (*raw genotyping data may be provided upon request*)

**Value of the data**

• Data presented here may be useful as an incentive for analyses of the SNPs rs7124442, rs10767664, rs962369, rs12273363 and rs908867, especially haplotypes consisting of two to five of these SNPs in larger samples from different populations with high suicide rates and also for comparative analyses of populations with high and low suicide rates.
• Supportive evidence to the proposed role of the SNP rs7124442 in the completed suicide phenotype [1] when in a specific allelic context (**A**/T–**T**/C–**T**/C–**C**/T) of at least two of the four SNPs (rs10767664, rs962369, rs12273363, rs908867).

## 1 Data

We provide data on additional analyses (Table 1, Table 2) of SNPs rs7124442, rs10767664, rs962369, rs12273363, rs908867 and rs1491851 for association with completed suicide phenotype, especially to highlight a proposed role of the SNP rs7124442 in the phenotype when in a specific haplotype context of two to four other SNPs (rs10767664, rs962369, rs12273363, rs908867) (Table 2). Raw genotyping data, potentially useful to conduct meta-analyses in the future, may be provided upon request.

## 2 Experimental design, materials and methods

Our study sample consisted of 775 unrelated Caucasian subjects, namely 289 controls (218 males and 71 females; mean age 51.9±18.3 years) and 486 suicide completers (362 males and 124 females; mean age 49.2±17.8 years). The control group was comprised of deceased persons, in which suicide as a cause of death was excluded. Criteria for positive alcohol dependence syndrome (ADS) status were met in 72 controls and in 97 suicide completers (for the details on the ADS status determination also see Materials and methods section in [1]).

Here, by means of software specifically designed for genetic analyses PLINK v1.07 [2], we performed logistic regression analyses (adjusted for covariates age, gender and ADS status) of the genotype data (raw file may be provided upon request) in order to gain more insight into the role of the studied SNPs in completed suicide phenotype [1].

First, the genotype analysis was done under four inheritance models: general genotype model (2 degrees of freedom), additive model (each copy of the minor allele alters the risk in an additive form), dominant model (a single copy of the minor allele is sufficient to modify the risk) and recessive model (two copies of the minor allele are needed to modify the risk) (Table 1). The data show no association of the studied SNPs under any of the model with completed suicide in our sample (*p*>0.05) (Table 1).

Haplotype analysis was carried out using 2-, 3- and 4-adjacent SNPs sliding window approach implemented in PLINK v1.07 [2]. We also carried out the haplotype analysis for all 5 SNPs (rs7124442, rs10767664, rs962369, rs12273363 and rs908867) which are in strong linkage disequilibrium (also see in Ref. [1, Fig. 1]). Haplotypes with frequencies lower than 1% were excluded from the analysis and correction for multiple testing was performed by 10,000 permutations test. As shown in Table 2, none of the haplotypes showed significant association with completed suicide after 10,000 permutations correction (EMP *p*>0.05). However, the T/C base change in the first position (rs7124442) in a specific context of common alleles (**A**/T–**T**/C–**T**/C–**C**/T) of at least two of the four SNPs rs10767664, rs962369, rs12273363 and rs908867, always resulted in at least a nominally significant *p*-value (Table 2).

## Figures and Tables

**Table 1:** Logistic regression analysis of the SNPs in the *BDNF* gene between controls and suicide completers, adjusted by age, gender and alcohol dependence syndrome status.

**SNP**	**Model**		**OR**	**95% CI**	***p***^a^
rs7124442	Geno_2df^b^	**TT***vs.* CT *vs.* CC	N/A	N/A	0.313
ADD^c^	2 CC+CT *vs.***TT**	1.234	0.939–1.623	0.131
Dominant^d^	**TT***vs.* CT+CC	1.106	0.821–1.489	0.508
Recessive^e^	**TT**+CT *vs.* CC	1.513	0.887–2.579	1.128
rs10767664	Geno_2df	**AA***vs.* TA *vs.* TT	N/A	N/A	0.161
ADD	2 TT+TA *vs.***AA**	1.296	0.954–1.761	0.097
Dominant	**AA***vs.* TA+TT	1.287	0.955–1.736	0.098
Recessive	**AA**+TA *vs.* TT	1.559	0.854–2.847	0.148
rs962369	Geno_2df	**TT***vs.* CT *vs.* CC	N/A	N/A	0.813
ADD	2 CC+CT *vs.***TT**	1.039	0.762–1.415	0.811
Dominant	**TT***vs.* CT+CC	0.940	0.698–1.267	0.686
Recessive	**TT**+CT *vs.* CC	1.113	0.606–2.045	0.730
rs12273363	Geno_2df	**TT***vs.* CT *vs.* CC	N/A	N/A	0.905
ADD	2 CC+CT *vs.***TT**	1.085	0.682–1.728	0.730
Dominant	**TT***vs.* CT+CC	0.977	0.706–1.352	0.889
Recessive	**TT**+CT *vs.* CC	1.191	0.472–3.008	0.711
rs908867	Geno_2df	**CC***vs.* TC *vs.* TT	N/A	N/A	0.838
ADD	2 TT+TC *vs.***CC**	0.956	0.464–1.970	0.902
Dominant	**CC***vs.* TC+TT	0.886	0.592–1.324	0.554
Recessive	**CC**+TC *vs.* TT	0.930	0.219–3.949	0.922
rs1491851	Geno_2df	**CC***vs.* TC *vs.* TT	N/A	N/A	0.623
ADD	2 TT+TC *vs.***CC**	0.994	0.813–1.216	0.953
Dominant	**CC***vs.* TC+TT	1.094	0.795–1.504	0.581
Recessive	**CC**+TC *vs.* TT	0.908	0.642–1.284	0.583

OR: odds ratio; CI: confidence interval.

a *p*-values are not corrected for multiple testing (Bonferroni); **bold**: major allele homozygotes.

b Geno_2df (general genotypic model): major allele homozygotes *vs.* heterozygotes *vs.* minor allele homozygotes.

c ADD (additive model, where each copy of the minor allele alters the risk in an additive form; a combination of the minor allele homozygotes with weight 2+heterozygotes is compared to major allele homozygotes): 2× minor allele homozygotes+heterozygotes *vs.* major allele homozygotes.

d Dominant: major allele homozygotes *vs.* heterozygotes+minor allele homozygotes.

e Recessive: major allele homozygotes+heterozygotes *vs.* minor allele homozygotes.

**Table 2:** Associations between *BDNF* gene haplotypes (3′→5′, reverse strand) and completed suicide (age, gender and alcohol dependence syndrome status adjusted).

**NSNP**	**NHAP**	**SNP-first**	**SNP2**	**SNP3**	**SNP4**	**SNP-last**	**Haplotype**	**Freq**	**OR**	**STAT**	***P***	**EMP*****p***
2	4	rs7124442				rs10767664	CT	0.0114	3.52	1.79	0.181	0.7516
2	4	rs7124442				rs10767664	TT	0.241	1.21	2.41	0.12	0.587
2	4	rs7124442				rs10767664	*CA*	0.255	1.1	0.651	0.42	0.9731
2	4	rs7124442				rs10767664	TA	0.493	0.794	4.98	**0.0256**⁎	0.1709

2	3	rs10767664				rs962369	AC	0.233	0.975	0.0399	0.842	1
2	3	rs10767664				rs962369	TT	0.245	1.25	3.22	0.0726	0.4028
2	3	rs10767664				rs962369	AT	0.514	0.858	2.14	0.144	0.6595

2	3	rs962369				rs12273363	CC	0.151	1.01	0.0063	0.937	1
2	3	rs962369				rs12273363	CT	0.0881	0.933	0.144	0.704	0.9997
2	3	rs962369				rs12273363	TT	0.756	1.03	0.0667	0.796	1

2	3	rs12273363				rs908867	TT	0.0824	0.9	0.317	0.573	0.9961
2	3	rs12273363				rs908867	CC	0.156	1.01	0.0033	0.954	1
2	3	rs12273363				rs908867	TC	0.762	1.04	0.102	0.749	1

3	4	rs7124442	rs10767664			rs962369	CAC	0.226	0.983	0.018	0.893	1
3	4	rs7124442	rs10767664			rs962369	TTT	0.24	1.22	2.57	0.109	0.5483
3	4	rs7124442	rs10767664			rs962369	CAT	0.0297	2.57	5.79	**0.0161**⁎	0.1124
3	4	rs7124442	rs10767664			rs962369	TAT	0.485	0.795	5.01	**0.0252**⁎	0.1689

3	4	rs10767664	rs962369			rs12273363	ACC	0.146	1	0.000101	0.992	1
3	4	rs10767664	rs962369			rs12273363	ACT	0.0851	0.918	0.211	0.646	0.9988
3	4	rs10767664	rs962369			rs12273363	TTT	0.245	1.26	3.44	0.0637	0.3651
3	4	rs10767664	rs962369			rs12273363	ATT	0.512	0.867	1.87	0.171	0.7323

3	3	rs962369	rs12273363			rs908867	CTT	0.0815	0.884	0.433	0.511	0.9895
3	3	rs962369	rs12273363			rs908867	CCC	0.151	1.02	0.0131	0.909	1
3	3	rs962369	rs12273363			rs908867	TTC	0.757	1.03	0.0417	0.838	1

4	5	rs7124442	rs10767664	rs962369		rs12273363	CACC	0.141	1.02	0.015	0.902	1
4	5	rs7124442	rs10767664	rs962369		rs12273363	CACT	0.0853	0.937	0.124	0.725	0.9999
4	5	rs7124442	rs10767664	rs962369		rs12273363	TTTT	0.24	1.22	2.57	0.109	0.5482
4	5	rs7124442	rs10767664	rs962369		rs12273363	CATT	0.0297	2.58	5.84	**0.0156**⁎	0.1098
4	5	rs7124442	rs10767664	rs962369		rs12273363	TATT	0.482	0.802	4.62	**0.0317**⁎	0.2031

4	4	rs10767664	rs962369	rs12273363		rs908867	ACTT	0.0803	0.876	0.496	0.481	0.9865
4	4	rs10767664	rs962369	rs12273363		rs908867	ACCC	0.147	1	2.77e-006	0.999	1
4	4	rs10767664	rs962369	rs12273363		rs908867	TTTC	0.245	1.24	3.06	0.0803	0.4357
4	4	rs10767664	rs962369	rs12273363		rs908867	ATTC	0.512	0.87	1.79	0.181	0.7511

5	5	rs7124442	rs10767664	rs962369	rs12273363	rs908867	CACTT	0.0794	0.86	0.642	0.423	0.9741
5	5	rs7124442	rs10767664	rs962369	rs12273363	rs908867	CACCC	0.141	1.01	0.0051	0.943	1
5	5	rs7124442	rs10767664	rs962369	rs12273363	rs908867	TTTTC	0.24	1.22	2.52	0.112	0.5603
5	5	rs7124442	rs10767664	rs962369	rs12273363	rs908867	CATTC	0.0302	2.66	6.19	**0.0129**⁎	0.09249
5	5	rs7124442	rs10767664	rs962369	rs12273363	rs908867	TATTC	0.482	0.803	4.55	**0.0328**⁎	0.21

NSNP: number of SNPs included in haplotype; NHAP: number of common haplotypes (haplotypes with Freq <0.01 were excluded from the analysis); SNP-first: SNP ID of the left-most (3′) SNP in the haplotype; SNP-last: SNP ID of the right-most (5′) SNP in the haplotype; Freq: frequency of a haplotype in the whole sample; OR: estimated odds ratio; STAT: test statistic (*T* from Wald test); *p*: asymptotic *p*-value.

⁎ Nominal significance; EMP *p*: empirical *p*-value after 10,000 permutations correction for multiple comparisons.
